# Supplementary material for: Multi‐dataset identification of innovative feature genes and molecular mechanisms in keratoconus
Source: J Cell Mol Med. 2024 Sep 19;28(18):e70079. doi: 10.1111/jcmm.70079 (PMC11412914; doi:10.1111/jcmm.70079)
Supplement: Supplementary file 2 — Material S2. [file JCMM-28-e70079-s002.zip › 7.GSEA analysis/3.DBNDD1/KEGG_CELL_ADHESION_MOLECULES_CAMS.html]

Details for gene set KEGG\_CELL\_ADHESION\_MOLECULES\_CAMS[GSEA]

|  || Dataset | DBNDD1.group.cls#h\_versus\_l |
| Phenotype | group.cls#h\_versus\_l |
| Upregulated in class | l |
| GeneSet | KEGG\_CELL\_ADHESION\_MOLECULES\_CAMS |
| Enrichment Score (ES) | -0.37395224 |
| Normalized Enrichment Score (NES) | -1.221791 |
| Nominal p-value | 0.16603054 |
| FDR q-value | 0.7114421 |
| FWER p-Value | 0.986 |
Table: GSEA Results Summary

  

Fig 1: Enrichment plot: KEGG\_CELL\_ADHESION\_MOLECULES\_CAMS      
 Profile of the Running ES Score & Positions of GeneSet Members on the Rank Ordered List

  

| SYMBOL | TITLE | RANK IN GENE LIST | RANK METRIC SCORE | RUNNING ES | CORE ENRICHMENT || 1 | ITGAL | na | 729 | 0.410 | 0.0002 | No |
| 2 | CD86 | na | 1030 | 0.379 | 0.0112 | No |
| 3 | PDCD1LG2 | na | 1052 | 0.376 | 0.0300 | No |
| 4 | CLDN5 | na | 1062 | 0.375 | 0.0492 | No |
| 5 | HLA-DRB5 | na | 1509 | 0.335 | 0.0537 | No |
| 6 | SELP | na | 1751 | 0.317 | 0.0632 | No |
| 7 | CTLA4 | na | 2265 | 0.283 | 0.0630 | No |
| 8 | CLDN22 | na | 2892 | 0.256 | 0.0582 | No |
| 9 | CD22 | na | 3242 | 0.241 | 0.0606 | No |
| 10 | SELPLG | na | 3365 | 0.237 | 0.0694 | No |
| 11 | PECAM1 | na | 4217 | 0.206 | 0.0554 | No |
| 12 | CLDN23 | na | 4736 | 0.189 | 0.0502 | No |
| 13 | CLDN9 | na | 5115 | 0.176 | 0.0484 | No |
| 14 | VCAN | na | 5694 | 0.158 | 0.0400 | No |
| 15 | ITGB2 | na | 6084 | 0.148 | 0.0364 | No |
| 16 | CD4 | na | 6459 | 0.138 | 0.0327 | No |
| 17 | PTPRM | na | 6543 | 0.136 | 0.0373 | No |
| 18 | CD8B | na | 6835 | 0.128 | 0.0356 | No |
| 19 | ITGA4 | na | 7459 | 0.114 | 0.0235 | No |
| 20 | CLDN19 | na | 7495 | 0.114 | 0.0284 | No |
| 21 | NRXN2 | na | 7693 | 0.110 | 0.0284 | No |
| 22 | CLDN20 | na | 7861 | 0.106 | 0.0291 | No |
| 23 | HLA-DMA | na | 8201 | 0.100 | 0.0244 | No |
| 24 | ICAM2 | na | 8229 | 0.099 | 0.0288 | No |
| 25 | CNTN1 | na | 8524 | 0.094 | 0.0252 | No |
| 26 | HLA-F | na | 8547 | 0.094 | 0.0294 | No |
| 27 | CLDN7 | na | 9113 | 0.084 | 0.0174 | No |
| 28 | MPZ | na | 9399 | 0.080 | 0.0133 | No |
| 29 | ICAM3 | na | 9736 | 0.075 | 0.0075 | No |
| 30 | ITGB7 | na | 9817 | 0.074 | 0.0090 | No |
| 31 | OCLN | na | 9867 | 0.073 | 0.0114 | No |
| 32 | CLDN11 | na | 9992 | 0.071 | 0.0115 | No |
| 33 | NLGN3 | na | 10091 | 0.070 | 0.0123 | No |
| 34 | HLA-DRB4 | na | 10303 | 0.067 | 0.0097 | No |
| 35 | ICOSLG | na | 10476 | 0.065 | 0.0080 | No |
| 36 | HLA-DRB1 | na | 10490 | 0.065 | 0.0110 | No |
| 37 | SDC2 | na | 10686 | 0.062 | 0.0086 | No |
| 38 | ITGAM | na | 11053 | 0.058 | 0.0010 | No |
| 39 | CNTN2 | na | 11583 | 0.051 | -0.0116 | No |
| 40 | HLA-DOA | na | 11835 | 0.049 | -0.0164 | No |
| 41 | PDCD1 | na | 12298 | 0.044 | -0.0274 | No |
| 42 | HLA-DQB1 | na | 12864 | 0.039 | -0.0417 | No |
| 43 | CLDN10 | na | 13201 | 0.036 | -0.0495 | No |
| 44 | CD34 | na | 13684 | 0.033 | -0.0618 | No |
| 45 | NLGN1 | na | 13768 | 0.032 | -0.0625 | No |
| 46 | NEGR1 | na | 13875 | 0.031 | -0.0640 | No |
| 47 | CNTNAP1 | na | 14665 | 0.025 | -0.0855 | No |
| 48 | CLDN16 | na | 15149 | 0.022 | -0.0983 | No |
| 49 | CLDN8 | na | 15262 | 0.021 | -0.1005 | No |
| 50 | MAG | na | 15661 | 0.018 | -0.1110 | No |
| 51 | NLGN4X | na | 15916 | 0.016 | -0.1175 | No |
| 52 | MPZL1 | na | 16080 | 0.015 | -0.1214 | No |
| 53 | CLDN3 | na | 16669 | 0.011 | -0.1378 | No |
| 54 | SDC1 | na | 16741 | 0.011 | -0.1393 | No |
| 55 | PTPRC | na | 16868 | 0.010 | -0.1424 | No |
| 56 | HLA-E | na | 17418 | 0.007 | -0.1579 | No |
| 57 | CDH1 | na | 18305 | 0.001 | -0.1835 | No |
| 58 | SDC3 | na | 18968 | -0.004 | -0.2024 | No |
| 59 | CD99 | na | 19357 | -0.007 | -0.2133 | No |
| 60 | ALCAM | na | 19413 | -0.008 | -0.2144 | No |
| 61 | CADM1 | na | 19611 | -0.010 | -0.2196 | No |
| 62 | HLA-G | na | 19797 | -0.011 | -0.2244 | No |
| 63 | HLA-C | na | 19946 | -0.013 | -0.2280 | No |
| 64 | CD58 | na | 20317 | -0.016 | -0.2379 | No |
| 65 | HLA-A | na | 20544 | -0.018 | -0.2435 | No |
| 66 | SDC4 | na | 20573 | -0.018 | -0.2434 | No |
| 67 | NRCAM | na | 20637 | -0.019 | -0.2443 | No |
| 68 | SELE | na | 20872 | -0.021 | -0.2500 | No |
| 69 | F11R | na | 20999 | -0.022 | -0.2524 | No |
| 70 | CLDN4 | na | 21176 | -0.024 | -0.2563 | No |
| 71 | HLA-DOB | na | 21192 | -0.024 | -0.2555 | No |
| 72 | ITGA9 | na | 21410 | -0.026 | -0.2604 | No |
| 73 | CD40 | na | 21562 | -0.028 | -0.2634 | No |
| 74 | CD28 | na | 21937 | -0.031 | -0.2726 | No |
| 75 | L1CAM | na | 21947 | -0.032 | -0.2712 | No |
| 76 | GLG1 | na | 23178 | -0.045 | -0.3044 | No |
| 77 | VCAM1 | na | 23198 | -0.046 | -0.3026 | No |
| 78 | PTPRF | na | 23791 | -0.054 | -0.3169 | No |
| 79 | HLA-B | na | 23866 | -0.054 | -0.3162 | No |
| 80 | CD276 | na | 24081 | -0.057 | -0.3194 | No |
| 81 | CDH5 | na | 24183 | -0.059 | -0.3193 | No |
| 82 | NLGN2 | na | 24244 | -0.060 | -0.3180 | No |
| 83 | CLDN1 | na | 24654 | -0.066 | -0.3264 | No |
| 84 | ICOS | na | 24698 | -0.066 | -0.3242 | No |
| 85 | CD8A | na | 24920 | -0.070 | -0.3270 | No |
| 86 | HLA-DRB3 | na | 25022 | -0.071 | -0.3262 | No |
| 87 | ITGAV | na | 25782 | -0.083 | -0.3438 | No |
| 88 | NEO1 | na | 26138 | -0.090 | -0.3494 | No |
| 89 | HLA-DRA | na | 26179 | -0.091 | -0.3458 | No |
| 90 | PVR | na | 26284 | -0.093 | -0.3440 | No |
| 91 | CD2 | na | 26358 | -0.095 | -0.3412 | No |
| 92 | CD274 | na | 26767 | -0.103 | -0.3476 | No |
| 93 | CDH2 | na | 26913 | -0.106 | -0.3464 | No |
| 94 | HLA-DMB | na | 26948 | -0.107 | -0.3418 | No |
| 95 | CD80 | na | 27049 | -0.109 | -0.3391 | No |
| 96 | NCAM2 | na | 27721 | -0.124 | -0.3520 | No |
| 97 | HLA-DPA1 | na | 28024 | -0.132 | -0.3539 | No |
| 98 | CNTNAP2 | na | 28718 | -0.149 | -0.3662 | Yes |
| 99 | ITGA6 | na | 28732 | -0.150 | -0.3588 | Yes |
| 100 | CLDN18 | na | 28929 | -0.155 | -0.3565 | Yes |
| 101 | ITGB1 | na | 29102 | -0.160 | -0.3532 | Yes |
| 102 | NFASC | na | 29252 | -0.164 | -0.3490 | Yes |
| 103 | CD6 | na | 29345 | -0.167 | -0.3430 | Yes |
| 104 | CDH3 | na | 29624 | -0.176 | -0.3418 | Yes |
| 105 | ESAM | na | 29649 | -0.177 | -0.3334 | Yes |
| 106 | CLDN6 | na | 30134 | -0.192 | -0.3374 | Yes |
| 107 | CLDN15 | na | 30342 | -0.199 | -0.3330 | Yes |
| 108 | ITGB8 | na | 30390 | -0.200 | -0.3240 | Yes |
| 109 | SIGLEC1 | na | 31308 | -0.233 | -0.3385 | Yes |
| 110 | CD40LG | na | 31492 | -0.240 | -0.3313 | Yes |
| 111 | CDH4 | na | 31512 | -0.241 | -0.3194 | Yes |
| 112 | ICAM1 | na | 32249 | -0.279 | -0.3262 | Yes |
| 113 | CDH15 | na | 32273 | -0.281 | -0.3123 | Yes |
| 114 | CADM3 | na | 32421 | -0.288 | -0.3016 | Yes |
| 115 | CLDN17 | na | 32713 | -0.307 | -0.2941 | Yes |
| 116 | SPN | na | 32832 | -0.316 | -0.2812 | Yes |
| 117 | JAM2 | na | 32852 | -0.317 | -0.2653 | Yes |
| 118 | HLA-DPB1 | na | 32889 | -0.320 | -0.2498 | Yes |
| 119 | NRXN3 | na | 33015 | -0.329 | -0.2363 | Yes |
| 120 | MADCAM1 | na | 33021 | -0.330 | -0.2194 | Yes |
| 121 | CLDN14 | na | 33082 | -0.334 | -0.2038 | Yes |
| 122 | CD226 | na | 33307 | -0.354 | -0.1919 | Yes |
| 123 | SELL | na | 33421 | -0.363 | -0.1763 | Yes |
| 124 | CLDN2 | na | 33855 | -0.411 | -0.1676 | Yes |
| 125 | NCAM1 | na | 33867 | -0.412 | -0.1465 | Yes |
| 126 | ITGA8 | na | 34117 | -0.445 | -0.1306 | Yes |
| 127 | JAM3 | na | 34561 | -0.588 | -0.1130 | Yes |
| 128 | NRXN1 | na | 34580 | -0.606 | -0.0821 | Yes |
| 129 | HLA-DQA2 | na | 34710 | -0.832 | -0.0427 | Yes |
| 130 | HLA-DQA1 | na | 34711 | -0.834 | 0.0005 | Yes |
Table: GSEA details [plain text format]

  

Fig 2: KEGG\_CELL\_ADHESION\_MOLECULES\_CAMS      
 Blue-Pink O' Gram in the Space of the Analyzed GeneSet

  

Fig 3: KEGG\_CELL\_ADHESION\_MOLECULES\_CAMS: Random ES distribution      
 Gene set null distribution of ES for **KEGG\_CELL\_ADHESION\_MOLECULES\_CAMS**
